# Volume, distribution and acidity of gastric secretion on and off proton pump inhibitor treatment: a randomized double-blind controlled study in patients with gastro-esophageal reflux disease (GERD) and healthy subjects

**DOI:** 10.1186/s12876-015-0343-x

**Published:** 2015-09-02

**Authors:** Andreas Steingoetter, Matthias Sauter, Jelena Curcic, Dian Liu, Dieter Menne, Michael Fried, Mark Fox, Werner Schwizer

**Affiliations:** 1 Division of Gastroenterology and Hepatology, University Hospital Zurich, Raemistrasse 100, CH-8091 Zurich, Switzerland; 2 Institute for Biomedical Engineering, University and ETH Zurich, Gloriastrasse 35, CH-8092 Zurich, Switzerland; 3 Menne Biomed Consulting, Olgastr. 7, D-72074 Tübingen, Germany

## Abstract

**Background:**

Postprandial accumulation of gastric secretions in the proximal stomach above the meal adjacent to the esophagogastric junction (EGJ), referred to as the ‘acid pocket’, has been proposed as a pathophysiological factor in gastro-esophageal reflux disease (GERD) and as a target for GERD treatment. This study assessed the effect of proton pump inhibitor (PPI) therapy on the volume, distribution and acidity of gastric secretions in GERD and healthy subjects (HS).

**Methods:**

A randomized, double blind, cross-over study in 12 HS and 12 GERD patients pre-treated with 40 mg pantoprazole (PPI) or placebo b.i.d. was performed. Postprandial secretion volume (SV), formation of a secretion layer and contact between the layer and the EGJ were quantified by Magnetic Resonance Imaging (MRI). Multi-channel pH-monitoring assessed intragastric pH.

**Results:**

A distinct layer of undiluted acid secretion was present on top of gastric contents in almost all participants on and off high-dose acid suppression. PPI reduced SV (193 ml to 100 ml, in HS, 227 ml to 94 ml in GERD; p < 0.01) and thickness of the acid layer (26 mm to 7 mm, 36 mm to 9 mm respectively, p < 0.01). No differences in secretion volume or layer thickness were observed between groups; however, off treatment, contact time between the secretion layer and EGJ was 2.6 times longer in GERD compared to HS (p = 0.012). This was not the case on PPI.

**Conclusions:**

MRI can visualize and quantify the volume and distribution dynamics of gastric secretions that form a layer in the proximal stomach after ingestion of a liquid meal. The secretion volume and the secretion layer on top of gastric contents is similar in GERD patients and HS; however contact between the layer of undiluted secretion and the EGJ is prolonged in patients. High dose PPI reduced secretion volume by about 50 % and reduced contact time between secretion and EGJ towards normal levels.

**Trial registration:**

NCT01212614.

**Electronic supplementary material:**

The online version of this article (doi:10.1186/s12876-015-0343-x) contains supplementary material, which is available to authorized users.

## Background

Postprandial accumulation of acid secretion in the proximal stomach above the meal adjacent to the esophagogastric junction (EGJ), referred to as the “acid pocket”, has been proposed as a pathophysiological factor in gastro-esophageal reflux disease (GERD) [1–4] and a target for GERD treatment [5–9]. The acid pocket is described in pH pull-through studies as a region of unbuffered gastric acid (>2pH drop to below pH 4) in close proximity to the EGJ [3]. This can be visualized also as a layer of gastric secretion in the proximal stomach on the surface of the meal by γ-scintigraphy and Magnetic Resonance Imaging (MRI) [10–13]. Studies combining pH-monitoring and γ-scintigraphy have shown that the acid pocket is the source of acid reflux early after meal ingestion [1, 7, 12]. Moreover the proximal border of the acid pocket has been shown to encroach on the EGJ in GERD patients such that acid secretions contact the distal esophageal mucosa. This phenomenon has been associated with the presence of reflux esophagitis and Barrett metaplasia [1–4].

One previous MRI study has shown that acid suppression by PPI reduces gastric content volume after meals in healthy volunteers [14]; however, simple volume measurements cannot differentiate between effects of PPI on gastric secretion and gastric emptying. Moreover if the acid pocket hypothesis is correct then it is not necessarily increased secretion volume but rather the abnormal distribution of unbuffered gastric secretions at the EGJ that increases the risk of acid reflux and mucosal disease in GERD patients.

The first objective of this study was to test the hypothesis that, compared to healthy subjects, there is abnormal distribution of gastric secretion in GERD patients that leads to prolonged contact between unbuffered acid and the EGJ after the meal. The second objective was to complete a randomized, placebo-controlled trial to document the effects of PPI on the volume, distribution and acidity of the gastric secretion layer and how this impacts on gastro-esophageal reflux after the meal.

## Methods

This clinical study was approved by the local ethics committee and registered at ClinicalTrial.gov (NCT01212614). Written informed consent was obtained prior to inclusion.

### Study design and test meal

**Fig. 1:**
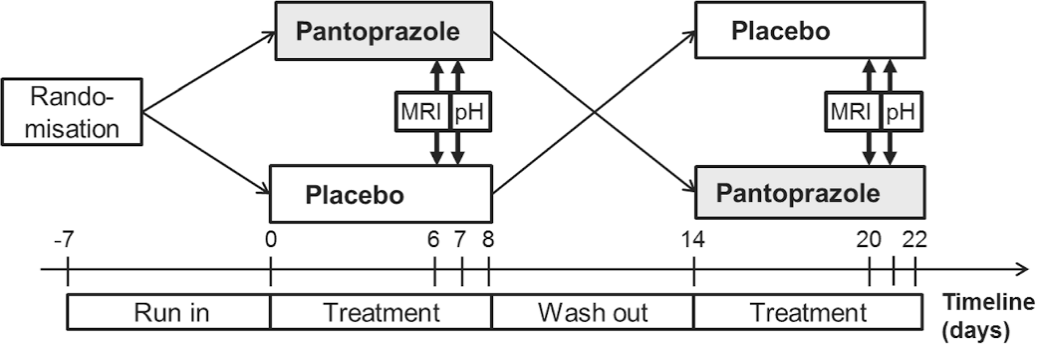
Flow chart of the double blind, crossover placebo controlled study

Healthy subjects (HS) and GERD patients without large hiatus hernia were investigated in a randomized, double blind, crossover, placebo controlled study performed at the University of Zurich and the Klinik Stephanshorn, St.Gallen, Switzerland, from December 2010 to December 2011. For study participant allocation, a computer-generated list of the study sequence was generated by an investigator with no clinical involvement in the trial (DM). Participants and investigators were blinded to randomization during data acquisition and analysis. The pantoprazole and placebo were in capsule form and identical in appearance. They were pre-packed in bottles and consecutively numbered for each participant according to the randomization schedule. The protocol (Fig. 1) included a MRI and pH-monitoring session in each study arm on consecutive days. Due to the MRI incompatibility of the pH monitoring device, pH studies were performed the day after MRI following the exact same time schedule of drug intake, test meal and body position. For GERD patients, there was a 7 day run-in phase without PPI or H_2_-Antagonists. Before each study session, subjects received one week of pantoprazole 40 mg b.i.d. or placebo. The two treatment arms were separated by a wash-out phase of 7-10 days. An antacid formula was provided (Riopan Gel®, Takeda Pharma AG, Switzerland) as rescue medication except for the examination days.

For each session, subjects arrived after a minimum 6 h fasting period at the study center. Study medication was taken and 45 to 60 min later subjects drank a secretion stimulating test meal in sitting position within 2 ± 1 min, The test meal was designed to promote gastric secretion and consisted of an acid stable, high caloric, viscous chocolate drink (400 ml, 450 kcal, pH 5.4) labelled with 167.5 μM of MR contrast agent (DOTAREM®) as described and validated previously [13].

### Population

Healthy, asymptomatic subjects (HS) with no history of digestive diseases and without reflux symptoms were recruited by advertisement. GERD patients were recruited from individuals referred for investigation of typical GERD symptoms (i.e. heartburn and/or regurgitation) of at least moderate severity (i.e. at least twice a week of at least moderate intensity) on the Eraflux questionnaire [15, 16]. GERD was diagnosed either by endoscopy confirming an erosive reflux disease (Stage A-C LA Classification) or pathological esophageal acid exposure in 24 h pH-monitoring performed within the previous 12 months (more than 4 % < pH4 over 24 h). None had history of upper gastrointestinal surgery. GERD patients were studied after being off acid suppressive medications for at least 7 days prior to study start. Exclusion criteria for both groups were prior abdominal surgery (except appendectomy, inguinal hernia repair), and intake of medication other than oral contraception. A Helicobacter-^13^C-breath test was performed to rule out Helicobacter pylori infection. High resolution esophageal manometry (HRM) was performed to rule out hiatal hernia > 3 cm or esophageal motility disorders and to measure the distance from the nares to the lower esophageal sphincter (LOS) for pH-probe placement.

### MRI measurements

Participants were imaged in right decubitus body position inside a 1.5 T clinical MRI System (1.5 T Achieva, Philips Medical Systems, The Netherlands) [13]. After a first MRI scan in fasting condition and the intake of the test meal, a MRI gastric volume scan followed by a validated T_1_-B_1_ mapping sequence (gastric secretion scan) were repeatedly performed at 10 min intervals until 120 min [17]. Details on the MRI sequence parameters of the gastric volume and secretion scans can be found in the Additional file 1.

### pH monitoring

Intragastric and esophageal pH were simultaneously measured using a 3 channel pH catheter consisting of three ISFET probes (UNISENSOR AG, Switzerland), connected to a pH recorder from MMS International (Medical Measurement Systems B.V., The Netherlands). The three gastric pH probes were located 3, 8 and 13 cm distal to the LOS, respectively. After placement of the pH catheter, the participants drank the test meal and spent 120 min in right decubitus position to copy the MRI session.

### Symptom scores

Symptoms were recorded before each MRI session. Heartburn, retrosternal pain, regurgitation and dysphagia were assessed and graded by their weekly and daily frequency, duration and intensity. The Eraflux score was applied to quantify the severity of GERD. Response was defined as an Eraflux score of <25 [15, 16].

### Data analysis

#### MRI meal and secretion volume

Gastric content volume (GCV), meal volume (MV) and secretion volume (SV) were derived by combining the gastric volume and secretion data according to previously reported procedures using in-house written software tools based on MATLAB 7.11 (The MathWorks, USA) and IDL (Exelis Visual Information Solutions, USA), respectively [13, 17].

In short, first the percentage of intragastric MV (*%meal*) from total GCV, measured in the gastric volume scans, was calculated for each time point using the exponential relationship between the T_1_ relaxation time, measured in the gastric secretion scans and the MR contrast agent concentration [*μM*] in the meal:

$$ \% meal=\frac{100\%}{167.5\mu M}\cdot \frac{1}{\beta}\cdot \ln \left(\frac{T_{10}}{T_1}\right), $$

with *β* = 0.013 μM^-1^ being the rate constant of the exponential relationship and *T*_*10*_ = 2.58 s being the T1 relaxation time of the test meal without MR contrast agent. The residual fasted content volume was negligible (17 ± 25 ml). Thus, MV = GCV **·** *%meal* and SV = GCV **-** MV were then calculated and plotted over time to generate the meal emptying and secretion curves. Secretion concentration was defined as *%secretion* = 100 - *%meal*. Meal emptying curves were fitted using the linear exponential (LinExp) gastric emptying model [18]. Meal half emptying time (t50), mean SV (meanSV) and maximum SV (maxSV) were determined.

**Fig. 2:**
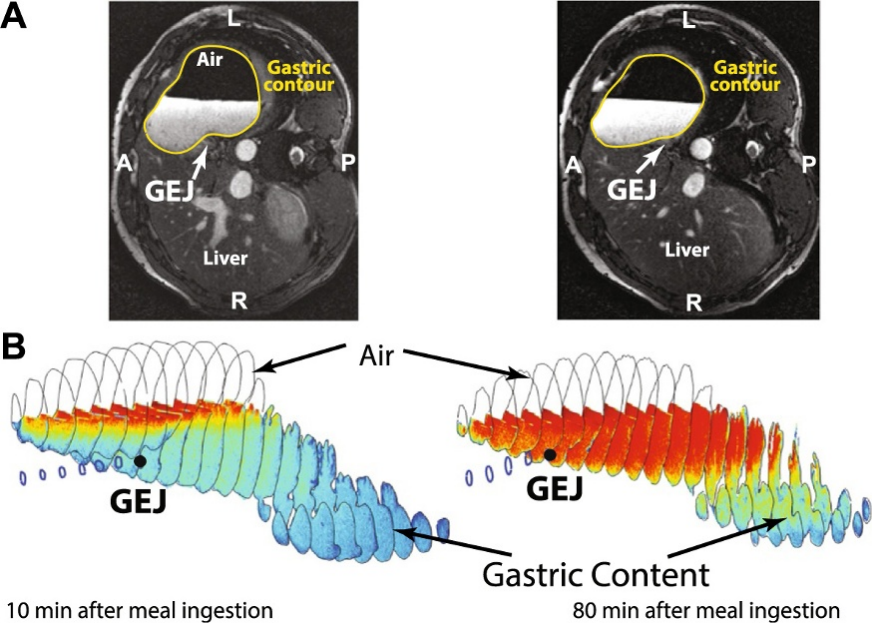
Definition of the EGJ position and 3D reconstruction of gastric content. **a** Transverse abdominal MR images at time points t = 10 and 80 min. EGJ is marked by a white arrow. Stomach contours are depicted as yellow lines. **b** The 3D reconstructed contours of stomach (black closed lines) and esophagus (dark blue closed lines) together with the color-coded gastric content (blue to red) of the same subject at the same time points. Gastric content was color-coded to highlight the formation of the secretion layer, here depicted in red. A non-linear color-coding of the contrast optimized grayscale values in the MR images was applied. The location of the EGJ is marked by the black dot at the end of the esophagus

To allow the calculation of the EGJ position relative to the acid secretion layer, the position of the EGJ in each gastric volume data set was extracted by selecting x-, y- and z-coordinates of the intercept of the mid-point of the sphincter mass with gastric content. This is illustrated for two different postprandial time points in the 2D MR images in Fig. 2a and the respective 3D reconstructions of stomach and gastric content in Fig. 2b.

### Gastric secretion layer

**Fig. 3:**
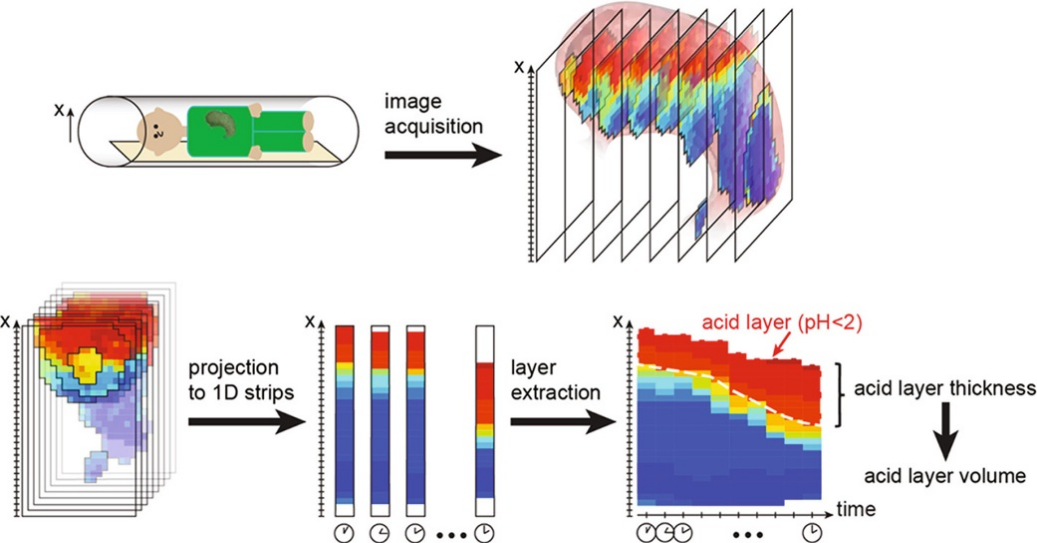
Schematic of post-processing steps for the quantification of acid layer thickness and volume from MR images. Gastric secretion accumulated predominantly on the meal surface and a secretion concentration gradient was observed from the surface of the meal into the test meal along the direction of gravity (i.e. the right side of the subject lying in the right lateral position) [10, 13]. The x-axis of the image data was set anti-parallel to the direction of gravity. Gastric content was sliced along the x-axis using a slice thickness of 1 mm and corresponding *%secretion* values were averaged along the other two Cartesian coordinates. This resulted in a 1D projection of mean *%secretion* values along the x-axis that was computed for each gastric secretion scan and stacked together over time to allow visualization of the formation of the gastric secretion layer (‘layer-graphs’). In the layer-graphs, the layer thickness was defined as the distance from the meal surface to the x coordinates having a threshold value of ≥70 % secretion. Layer volume (LV) was calculated by summing all pixels above this threshold

The volume and thickness of the gastric secretion layer was extracted from the dedicated gastric secretion scans. The layer was defined as a distinct region in the proximal stomach on top of the gastric contents with *%secretion* of ≥ 70 % (i.e. *%meal* of < 30 %). The 70 % secretion threshold was chosen on the basis of *in vitro* experiments. Serial dilutions of the test meal were performed with 0.1 N HCl^-^ as a simple surrogate for gastric acid secretions [17]. These experiments determined that *%secretion* of ≥ 70 % (i.e. *%meal* of < 30 %) resulted in pH ≤2 in the meal-secretion mixture. This conservative pH threshold is within that applied in studies that used the pH-sensor pull-through method to define the extent of the acid pocket. Very similar results were obtained in previous studies by titration with gastric secretions obtained at endoscopy [10, 17]. Image processing steps for layer quantification are depicted and explained in Fig. 3.

### Contact time between gastric secretion layer and esophagogastric junction (EGJ)

Layer-graphs (Fig. 7) were produced that integrate information on the formation and position of the gastric secretion layer from secretion scans and the EGJ position from anatomical MRI scans (Figs. 2 and 3). The contact time between the gastric secretion layer *%secretion* of ≥70 % (i.e. *%meal* of < 30 %) was calculated for each subject. The time to first occurrence of ≥70 % secretion at the EGJ was then subtracted from the maximum recording time of 120 min to obtain the absolute period of contact time. This result was normalized to the subject’s t50 to provide an additional assessment of contact time that was independent of emptying dynamics:

$$ Relative\_EGJ\_ contact\_ timetoLayer\kern0.75em =\kern0.2em \left(120\  min\kern0.5em \hbox{--} \kern0.5em  timetofirstLayerformation\right)/t50 $$

This metric describes the contact time of the EGJ with the secretion layer relative to the gastric half emptying time (t50). Thus, values >1 indicate that the secretion layer was in contact for more than half the time taken to empty the meal from the stomach. In cases where the EGJ was not exposed to the gastric secretion layer, the time to first occurrence of ≥70 % secretion was set to the maximum recording time.

### pH monitoring

Data from pH monitoring were visually inspected for data quality by MS and WS using the MMS International software tool (Medical Measurement Systems B.V., The Netherlands). Raw data were extracted and analyzed with home-built algorithms implemented in program R (R Foundation for Statistical Computing, Austria). From the three intragastric pH- probes, lowest intragastric pH (mingastricpH) was extracted and median mingastricpH over two hours postprandial were calculated.

### Symptom scores

The severity and frequency of reflux symptoms reported by participants were calculated using the publicly available Eraflux score calculator (http://www.menne-biomed.de/biomed/erascore2.html) [16].

### Statistics

Statistical analyses were performed using program R 2.15./RStudio 0.98.274. The LinExp model was computed by population non-linear mixed effect modelling using function *nlme* [19]. T50 values were numerically derived from parameters *κ* and *t*_*empt*_ as described previously [18]. Univariate linear mixed model analysis using function *lme* was applied to evaluate the effects of study group and treatment on continuous study parameters. Eraflux scores in GERD patients under placebo and PPI were compared by a linear model using function *lm* in program R. Correlation between SV and LV was performed by linear regression also using function *lm*. Parameter estimates are presented as mean ± standard error and in case of Poisson distributed data as mean (95 % confidence interval).

## Results

### Participants

Twelve asymptomatic HS (8 m/4w, age 26 ± 8 years, BMI 23 ± 2 kg/m^2^) and twelve GERD patients (7 m/5w, 45 ± 11, BMI 24 ± 3 kg/m^2^) were recruited and all completed the study. MRI secretion data from the PPI arm of two GERD patients and pH data from one HS were discarded due to technical difficulties. Estimates ± SE for all investigated parameters are summarized in Table 1.

**Table 1:** Primary and secondary outcome parameter results (data as estimate ± standard error from the *lme* model)

Parameter	Modality	HS		GERD	
		Placebo	PPI	Placebo	PPI
mean SV [ml]	MRI	133 ± 14	73 ± 14*	152 ± 14	66 ± 14*
max SV [ml]	MRI	193 ± 17	100 ± 18*	227 ± 18	94 ± 17*
Layer thickness at 60 min [mm]	MRI	26 ± 5	7 ± 5*	36 ± 5	9 ± 5*
Layer Volume at 60 min [ml]	MRI	79 ± 16	20 ± 16*	117 ± 17	18 ± 17*
Median mingastricph	pH-monitoring	1.8 ± 0.3	4.5 ± 0.3*	1.5 ± 0.3	3.8 ± 0.3*
2 h postprandial					
t50 [min]	MRI	60 ± 6	71 ± 6	62 ± 6	64 ± 6
EGJ exposure time to ≥ 70 % secretion layer Normalized to T50	MRI	0.45 ± 0.2	0.42 ± 0.2	1.18 ± 0.2***	0.42 ± 0.2**
Eraflux symptoms score	Questionnaire	0 ± 0	0 ± 0	33 ± 3	20 ± 3*

* <0.05, ** *p* < 0.01 different to placebo

*** <0.05, *p* < 0.01 different to HS

Poisson distributed parameter

### MRI meal and secretion volumes

**Fig. 4:**
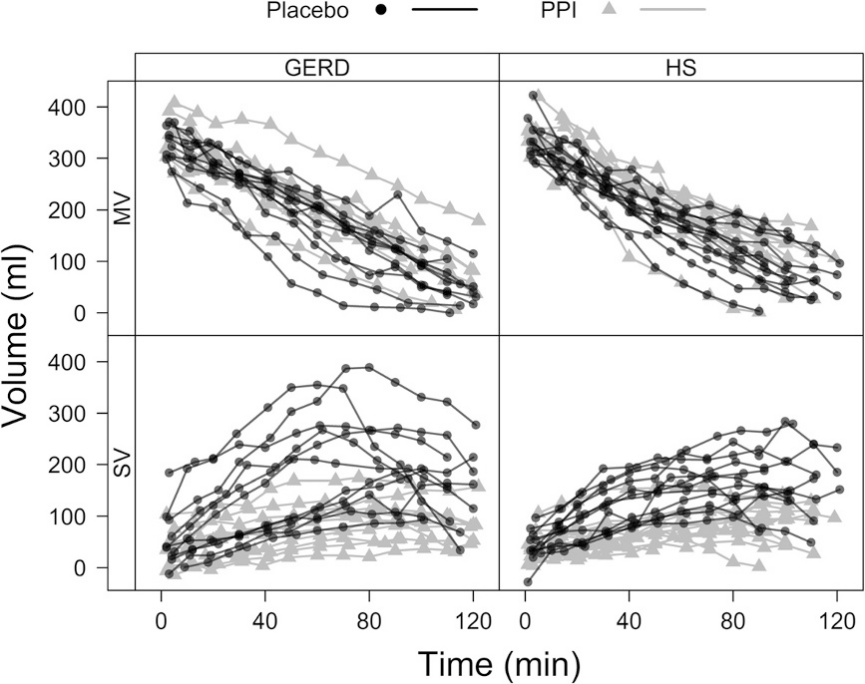
Grouped meal and secretion volume curves. Meal Volume (MV) and Secretion Volume (SV) dynamics over 120 min for GERD patients and Healthy Subjects (HS), under placebo (black dots/lines) and PPI (grey triangles/lines). The approximately linear emptying pattern of the test meal is typical for a high calorie, viscous liquid meal

Overall maxSV was approximately 200 ml in both groups under placebo (Table 1) with no difference between groups. PPI treatment reduced maxSV by 93 ± 20 ml in HS and 134 ± 20 ml in GERD patients, respectively. No difference was observed between study groups in either study arm, all p ≥ 0.2. Individual MV and SV curves presented by study group and treatment are plotted in Fig. 4. Meal emptying, as assessed by t50, was similar in both study groups (HS vs. GERD) and was not significantly altered by treatment (placebo vs. PPI), all p > 0.2.

### Gastric secretion layer

**Fig. 5:**
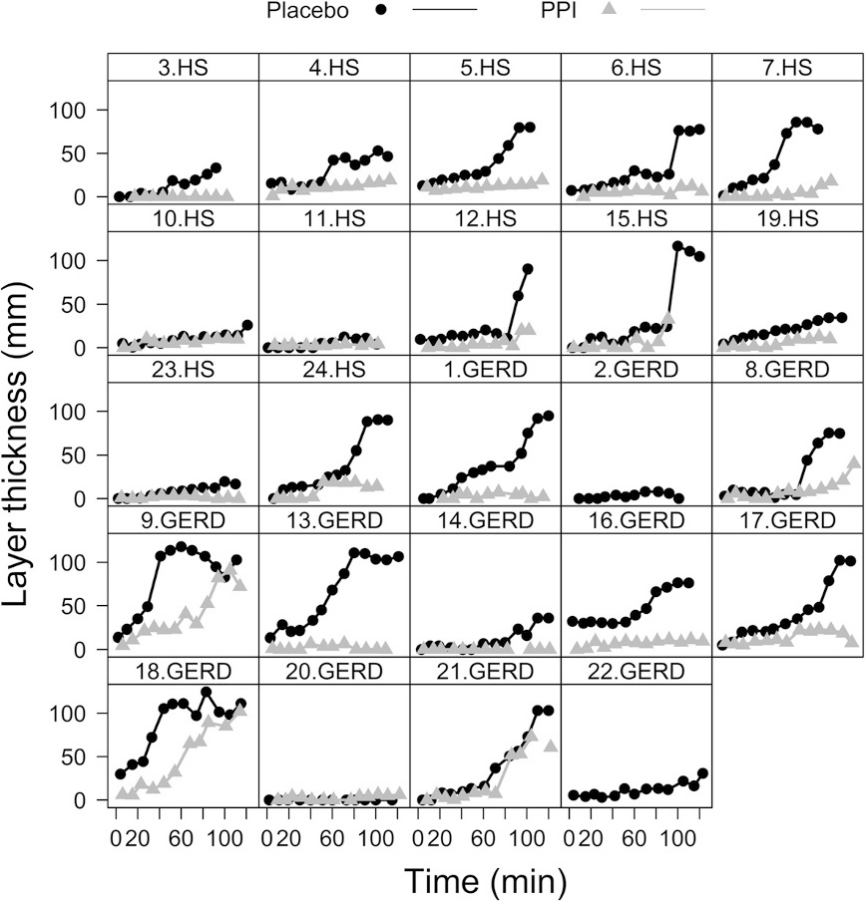
Formation of individual secretion layers. Layer thickness over time is depicted for all healthy subjects (HS) and GERD patients under placebo (black dots/lines) and PPI therapy (grey triangles/lines)

On placebo, gastric secretion accumulated in the proximal stomach forming a distinct secretion layer on top of the meal in 23 of 24 subjects. Variability in secretion layer volume and thickness between subjects was large in both study groups. On PPI, the distinct secretion layer persisted in all but two HS and one GERD patient. Layer formation in each subject for both treatment arms is depicted in Fig. 5.

**Fig. 6:**
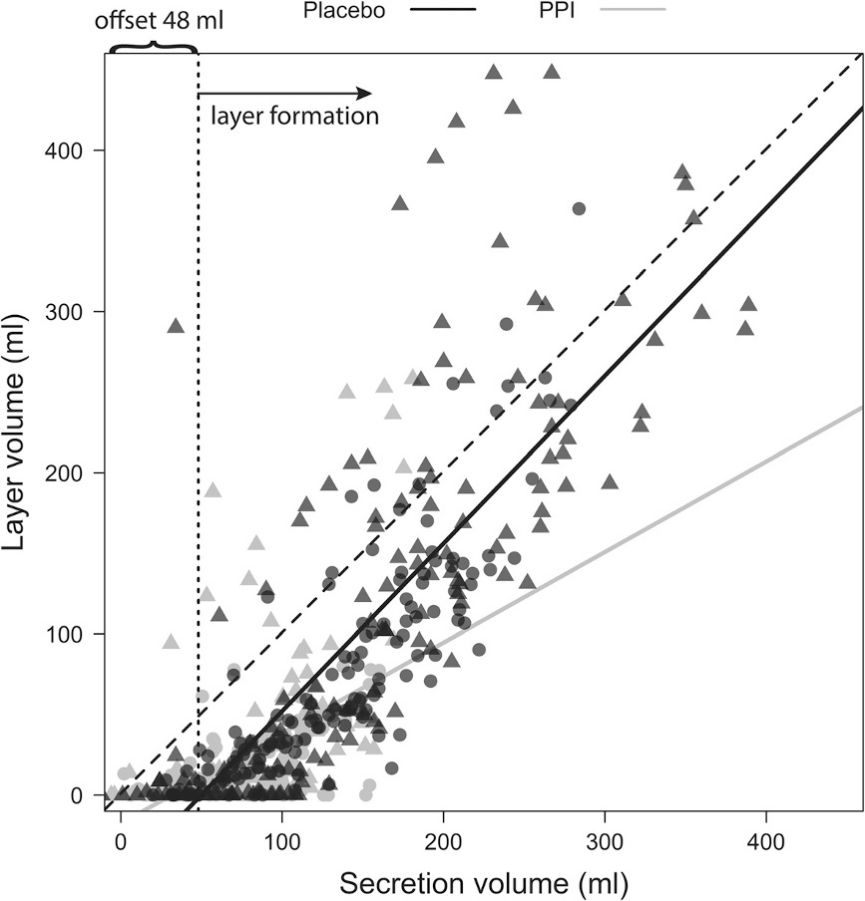
Correlation of layer and secretion volume under placebo and PPI therapy. GERD and HS data are depicted as triangles and circles, respectively; placebo volumes (black), PPI volumes (grey). The offset in the correlation indicates that layer formation started when approximately 50 ml secretion was present in the stomach. For placebo, the regression coefficient was R^2^ = 0.7 with regression slope and offset of 1.04 ± 0.04 and -52 ± 7 ml, respectively. On pantoprazole therapy, the regression coefficient was reduced to R^2^ = 0.3 with regression slope and offset of 0.56 ± 0.06 and offset -18 ± 5, respectively

Layer thickness at 60 min after the meal decreased on PPI treatment by 19 ± 1.6 mm for HS and 27 ± 1.7 mm for GERD (p < 0.0001 in both groups), with no difference between the study groups (both p ≥ 0.4). On placebo there was a positive linear correlation between layer volume and secretion volume (Fig. 6). The secretion layer (defined as ≥70 % secretions) appeared when approximately 50 ml of secretion accumulated in the stomach and then increased in direct proportion with overall secretion volume.

### Contact time between gastric secretion layer and esophagogastric junction (EGJ)

**Fig. 7:**
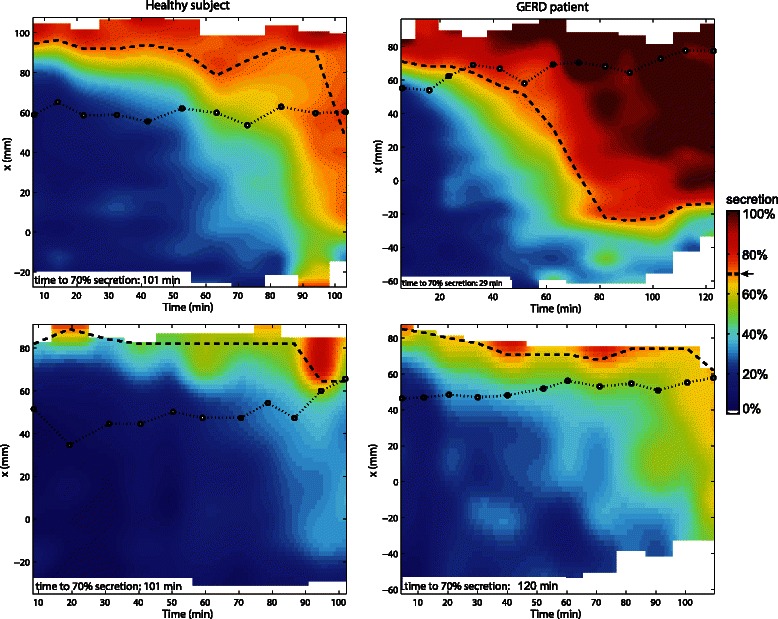
Layer graphs including EGJ position and time to Layer formation. Layer graphs of a healthy subject (left) and a GERD patient (right) under placebo (top) and PPI therapy (bottom). The vertical axis represents the MR image x-axis, which is aligned along the direction of gravity. The colours code the average *%secretion*. The threshold level of the layer (i.e. ≥70 % secretion) and the position of the EGJ are indicated as white dashed and black dotted line, respectively

The layer-graphs of one healthy subject and one GERD patient during the placebo and pantoprazole study arm showing the EGJ position relative to the gastric secretion layer are depicted in Fig. 7. For placebo, normalized EGJ contact time to the layer (≥70 % secretion) relative to gastric half emptying time (t50) was higher in GERD patients than HS (p = 0.012, Table 1). This indicates that off medication the EGJ was in contact with the gastric secretion layer for considerably more than half the gastric half emptying time in GERD patients, but for only about half of the half emptying time in HS. Absolute exposure times were 34 ± 9 for HS and 55 ± 9 min for GERD on placebo. PPI treatment reduced the normalized contact time to the layer in in GERD patients (p = 0.03) and comparison between groups on acid suppression no longer showed significant differences (p = 0.9). Absolute exposure times on PPI were 21 ± 9 for HS and 28 ± 9 min for GERD, respectively.

### pH monitoring data

MingastricpH during the 2 h after the meal in HS and GERD patients was pH 1.8 ± 0.3 and pH 1.5 ± 0.3, respectively. MingastricpH during the 2 h postprandial period increased under PPI in HS and GERD patients by pH 2.7 ± 0.4 and pH 2.3 ± 0.3, respectively. No difference was detected between study groups (placebo: p = 0.5, PPI: p = 0.1).

### Symptom scores

Symptoms improved significantly under PPI in GERD patients. Eraflux symptom scores decreased by 13 ± 4 (p = 0.003) with 8 of 12 patients reporting a response, defined as an Eraflux symptom score under PPI below a score of 25 (i.e. one or less symptoms per week of only weak intensity).

## Discussion

This study applied Magnetic Resonance Imaging (MRI) methodology to visualize and quantify the volume and distribution of gastric secretions that form a layer in the proximal stomach after ingestion of a liquid meal. There was no difference in layer formation, secretion volume, intragastric pH, or gastric emptying between healthy subjects and gastro-esophageal reflux disease (GERD) patients on placebo. However MRI demonstrated that the distribution of gastric secretions in the stomach was abnormal in the patient group such that the exposure of the EGJ to undiluted gastric secretion was significantly more prolonged than in healthy controls. High dose proton pump inhibitor (PPI) therapy reduced secretion volume, layer thickness and intra-gastric acidity in both healthy subjects and GERD patients; however a persistent layer of gastric secretion was still present in the majority of individuals on acid suppression.

The first objective of this study was to test the hypothesis that, compared to healthy subjects, there is either increased volume or abnormal distribution of gastric secretion in GERD patients. The results demonstrate that collection of undiluted gastric secretion in the proximal stomach after a meal, a key component of the “acid pocket”, is a normal physiological phenomenon that occurs in both healthy subjects and GERD patients [1–3, 20]. The thickness of the layer documented by MRI was very comparable to the length of the acid pocket after ingestion of a refluxogenic meal on pH pull-through studies [21]. No differences were observed between the study groups with regard to layer formation, total gastric secretion volume or gastric emptying. However, off treatment, the period of time that the EGJ was in contact with the undiluted layer of gastric secretion was more than twice as long in GERD patients as healthy subjects. This confirms that it is not the volume of gastric secretions *per se* but rather the distribution of secretions within the proximal stomach that is abnormal in GERD patients and that leads to prolonged exposure of the EGJ to gastric secretions after the meal [12]. It is clear that the proximity of undiluted gastric secretions to a weak, mechanically insufficient reflux barrier in such patients will increase the risk of acid reflux. Consistent with this view, pH-studies have shown that the presence of unbuffered gastric acid adjacent to the EGJ is more important than the size (i.e. length) of the “acid pocket” in determining the risk of reflux [1, 2]; however, in the absence of an hiatus hernia, these studies could not explain why this occurred. As the volume of secretions is the same in both groups, abnormal distribution of gastric secretions within the stomach must reflect abnormal morphology of the EGJ and proximal stomach in GERD patients. The three-dimensional structure and function of this region in GERD patients has been studied by concurrent MRI and high resolution manometry [22, 23]. This work demonstrated that the esophagogastric insertion angle was obtuse, the insertion of the esophagus into the stomach and the orientation of the proximal stomach within the abdomen are altered in GERD patients even in the absence of hiatus hernia [24, 25]. The effect of gastric accommodation or motility on the proximal distribution of gastric secretion was not assessed, however, recent papers provide little evidence that accommodation of the stomach (or intra-gastric pressure) is different in health and GERD [24, 26].

The second objective was to document the effects of high dose acid suppression by proton pump inhibitors on the volume, distribution and acidity of the gastric secretion layer. The findings of the randomized, placebo-controlled trial demonstrate that after one week of treatment with 40 mg b.i.d. pantoprazole, gastric secretion volume was reduced approximately by 50 % compared to placebo (Table 1). The reduction in the volume and thickness of the layer of gastric secretion above the meal and contact time with the EGJ was even more marked, approximately 75 % in both groups. However a distinct layer of undiluted secretion was still visible in almost all subjects despite high dose PPI treatment (Table 1). A recent γ-scintigraphy study in GERD patients quantified a reduction in the pooling of ^99m^Tc-pertechnetate (a surrogate for chloride ions) of 43 % and a reduction in the cross-sectional area of the acid pocket of 33 % [12]. The larger effects on the acid layer observed in this study can be logically explained by the three-dimensional spatial information extracted from MRI data, compared to the two-dimensional projection information obtained from γ-scintigraphy.

Intra-gastric pH monitoring documented that the acidity of the residual layer increased from below pH 2 on placebo to pH 4.5 ± 0.3 and 3.8 ± 0.3 on PPI in healthy subjects and GERD patients, respectively. This is very similar to the results of previous pH pull-through studies that documented the effects of PPI on the “acid pocket” by pH pull-through in healthy subjects and GERD patients [21, 27]. In summary, this study makes clear that the effect of high-dose PPI on acid production is at least an order of magnitude greater than its effects on gastric secretion volume (i.e. ~1 % ongoing secretion of protons (increase of 2 pH units) vs. ~50 % production of gastric secretion volume on PPI). These findings indicate that suppression of gastric secretion is incomplete and layering of undiluted secretion persists under PPI therapy. Although a recent pH pull-through and γ-scintigraphy study found no difference in the position or the pH of the acid pocket between partial and complete PPI responders [27], reasons for symptom persistence on PPI are varied, and the persistent layer of secretions adjacent to the EGJ seen on MRI provides a ready source of mildly acid reflux (~pH4) after meals [1, 12]. In this situation the therapeutic aims of GERD patients with persistent symptoms on acid suppression should not be limited to increasing intra-gastric pH by further increasing PPI dose. Rather supplementary therapy that involves displacing the layer of secretions away from the EGJ as described in recent studies with raft-forming alginates such as Gaviscon Advance (Reckitt Benckiser, Slough, UK) may provide additional protection from symptomatic reflux from the “acid” or, on PPI, the “mildly-acid” pocket [5, 6, 9, 28].

This study demonstrates that combining MRI allows non-invasive assessment of intragastric meal, secretion and pH distribution at high spatial and temporal resolution. Applied after ingestion of a secretion stimulating test meal, this approach represents an efficient tool to assess the efficacy of pharmaceutical agents and their potential effect on gastric motor and secretory function in general and the “acid pocket” in particular. Our MRI method to assess intragastric secretion meal volumes has been evaluated and applied in previous studies [10, 13, 17, 18, 29, 30], and has been expanded in this work by the quantification of the secretion layer and its influence by PPI therapy. The unique advantage of this approach is that the distribution and layering of gastric secretion can be visualized and analyzed locally in three-dimensions and directly related to gastric and gastro-esophageal structure and function. Limitations of this technique include the need to study patients lying down in the MRI scanner rather than in the upright position. The location of the gastric secretion layer in the stomach and, thus, EGJ contact time with the layer both vary with body position [31, 32]. This may be due to the morphological changes at the EGJ that favor collection of gastric secretions in this region [29]. Further, current catheter and sensor technology for the measurement of intragastric pH are not MRI compatible and, thus, required a sequential rather than a combined measurement protocol for MRI and pH-metry. Another issue is that liquid nutrients do not represent a “normal meal”; however, this is necessary to allow estimates of secretion volume and distribution of secretion within the stomach. Note that a previous study demonstrated no difference in acid reflux events between liquid and solid meals with identical nutritional composition [33]. A limitation of the T1 mapping method applied to document secretion is that it does not differentiate between gastric, duodenal or saliva secretion (all exhibit similar T1 values) [13]. To simulate dilution of the labelled test meal by gastric secretion and to correlate meal dilution with intra-gastric pH, an *ex vivo* titration experiment was performed using 0.1 N HCl^-^. A meal dilution of ≥ 70 % gastric secretion per image voxel, which reflected a pH threshold of pH ≤ 2 off PPI, was defined as the secretion layer. Note that, *in vivo,* the secretion layer can additionally be buffered by substances such as saliva and mucus. Otherwise, the layer pH can also contain digestive products such as amino acids and free fatty acids and also be contaminated by bile acids. Since simulated gastric fluid including all mentioned factors is not yet available for *in vitro* digestion [34], the extent by how much these factors may have altered the actual pH value in the layer remains speculative. Nevertheless, the MRI approximated pH values were comparable to the actual intragastric pH documented on sequential pH studies. An application of the conventional threshold of pH ≤ 4 (equivalent to a dilution of ≥17 % secretion) used to define the acid pocket in pH pull-through studies would have had resulted in even larger estimates of intragastric secretion volume and layer thickness.

## Conclusions

This study confirms that a layer of gastric secretion on the surface of gastric contents is always present after meals. The volume of gastric secretion was very similar in healthy subjects and GERD patients; however the distribution of gastric secretions in the stomach was abnormal in GERD patients and resulted in prolonged exposure of the EGJ to unbuffered gastric secretions. This improved on 40 mg pantoprazole b.i.d. but layering of mildly acid secretion at the EGJ persisted in most individuals and this could well represent the source of mildly acid reflux that persists in some patients on high dose PPI treatment. MRI with T1 mapping represents a powerful non-invasive tool for further investigations on therapies targeting the reduction of gastric secretion and reflux from the “acid pocket”.

## Additional file

Additional file 1: **Details on MRI sequence parameters.** MRI sequence parameters of the gastric volume scan were: Steady state free precession sequence (b-FFE); 30 axial image slices; slice thickness = 6 mm; field of view = 360 mm; scan matrix = 240 × 192; repetition time = 3.3 msec; echo time = 1.5 msec; flip angle = 60°; scan time = 15.5 s, one breath hold. MRI sequence parameters of the T1-B1 mapping sequence (gastric secretion scan) were for T1 mapping: Dual flip angle gradient echo sequence, 8 axial image slices, slice thickness = 15 mm, slice gap = 0.5 mm, field of view = 360 mm, scan matrix = 128 × 128, repetition time = 9 msec, echo time = 3.6 msec, flip angles = 5° and 31°, number of dummy excitations = 29 and 21, scan time = 15 s, one breath hold. For B1 mapping: Dual repetition time gradient echo sequence, slice thickness = 15 mm, slice gap = 0.5 mm, flip angle = 70°, field of view = 360 mm, scan matrix = 64 × 64, repetition time 1 (TR1) = 20 msec, repetition time 2 = 100 msec, echo time = 3.6 msec, number of dummy excitations = 6, scan time = 54 s, three breath holds. (PDF 70 kb)
