# Supplementary material for: Volume, distribution and acidity of gastric secretion on and off proton pump inhibitor treatment: a randomized double-blind controlled study in patients with gastro-esophageal reflux disease (GERD) and healthy subjects
Source: BMC Gastroenterol. 2015 Sep 2;15:111. doi: 10.1186/s12876-015-0343-x (PMC4557316; doi:10.1186/s12876-015-0343-x)
Supplement: Additional file 1: — Details on MRI sequence parameters. MRI sequence parameters of the gastric volume scan were: Steady state free precession sequence (b-FFE); 30 axial image slices; slice thickness = 6 mm; field of view = 360 mm; scan matrix = 240 × 192; repetition time = 3.3 msec; echo time = 1.5 msec; flip angle = 60°; scan time = 15.5 s, one breath hold. MRI sequence parameters of the T1-B1 mapping sequence (gastric secretion scan) were for T1 mapping: Dual flip angle gradient echo sequence, 8 axial image slices, slice thickness = 15 mm, slice gap = 0.5 mm, field of view = 360 mm, scan matrix = 128 × 128, repetition time = 9 msec, echo time = 3.6 msec, flip angles = 5° and 31°, number of dummy excitations = 29 and 21, scan time = 15 s, one breath hold. For B1 mapping: Dual repetition time gradient echo sequence, slice thickness = 15 mm, slice gap = 0.5 mm, flip angle = 70°, field of view = 360 mm, scan matrix = 64 × 64, repetition time 1 (TR1) = 20 msec, repetition time 2 = 100 msec, echo time = 3.6 msec, number of dummy excitations = 6, scan time = 54 s, three breath holds. (PDF 70 kb) [file 12876_2015_343_MOESM1_ESM.pdf]

Details on MRI sequence parameters

MRI sequence parameters of the gastric volume scan were: Steady state free precession sequence (b-FFE); 30 axial image slices; slice thickness = 6 mm; field of view = 360 mm; scan matrix = 240 x 192; repetition time = 3.3 msec; echo time = 1.5 msec; flip angle = 60°; scan time = 15.5 sec, one breath hold.

MRI sequence parameters of the T_1-B_1 mapping sequence (gastric secretion scan) were for T1 mapping: Dual flip angle gradient echo sequence, 8 axial image slices, slice thickness = 15 mm, slice gap = 0.5 mm, field of view = 360 mm, scan matrix = 128 x 128, repetition time = 9 msec, echo time = 3.6 msec, flip angles = 5° and 31°, number of dummy excitations = 29 and 21, scan time = 15 sec, one breath hold.

For B_1 mapping: Dual repetition time gradient echo sequence, slice thickness = 15 mm, slice gap = 0.5 mm, flip angle = 70°, field of view = 360 mm, scan matrix = 64 x 64, repetition time 1 (TR1) = 20 msec, repetition time 2 = 100 msec, echo time = 3.6 msec, number of dummy excitations = 6, scan time = 54 sec, three breath holds.
